# Problems in the reporting of acne clinical trials: a spot check from the 2009 Annual Evidence Update on Acne Vulgaris

**DOI:** 10.1186/1745-6215-11-77

**Published:** 2010-07-12

**Authors:** John R Ingram, Douglas JC Grindlay, Hywel C Williams

**Affiliations:** 1 Welsh Institute of Dermatology, University Hospital of Wales, Heath Park, Cardiff, Wales, CF14 4XW, UK; 2 NHS Evidence - skin disorders, Centre of Evidence Based Dermatology, University of Nottingham, NG7 2NR, UK

## Abstract

In the course of producing the 2009 NHS Evidence - skin disorders Annual Evidence Update on Acne Vulgaris, 25 randomised controlled trials were examined. From these, at least 12 potentially serious problems of trial reporting were identified. Several trials concluded no effect of a treatment yet they were insufficiently powered to exclude potentially useful benefits. There were examples of duplicate publication and "salami publication", as well as two trials being combined and reported as one. In some cases, an incorrect "within-groups" statistical comparison was made and one trial report omitted original efficacy data and included only P values. Both of the non-inferiority studies examined failed to pre-specify a non-inferiority margin. Trials reported as "double-blind" compared treatments that were dissimilar in appearance or had differing adverse effect profiles. In one case an intention-to-treat analysis was not performed and there was a failure to account for all of the randomized participants. Trial results were made to sound more impressive by selective outcome reporting, emphasizing the statistical significance of treatment effects that were clinically insignificant, and by the use of larger-sounding odds ratios rather than rate ratios for common events. Most of the reporting problems could have been avoided by use of the CONSORT guidelines and prospective trial registration on a public clinical trials database.

## Introduction

Each year, *NHS Evidence - skin disorders *(a national specialist library funded by NICE, available at http://www.library.nhs.uk/skin) publishes an Annual Evidence Update on Acne Vulgaris, which is a search for new evidence published or indexed in the last year [1]. NHS Evidence - skin disorders also produces Annual Evidence Updates on atopic eczema, psoriasis and skin cancer. The purpose is to make our community of clinical users (mainly dermatologists, general practitioners and nurses) aware of newly published research studies, to discuss their significance for clinical practice, and to warn of any methodological issues in their interpretation.

The Annual Evidence Updates normally search for systematic reviews and guidelines, because of the potential hazards in commenting on single randomized controlled trials or RCTs [2]. However, as only one systematic review on acne was found for our 2009 Annual Evidence Update, which was published on 2^nd ^March 2009, we also searched for new RCTs published or indexed over the previous year since the last Annual Evidence Update [1,3]. A full description of the methodology and search strategies used can be found on the Annual Evidence Update web pages [1].

The RCTs that were found for the 2009 Annual Evidence Update comprise a "spot check" of acne trials published over a one year period. In the course of putting together the Annual Evidence Update [1,3] the authors were struck by a high frequency of problems in the reporting and interpretation of these acne RCTs, which are now highlighted in this article. Our perspective in this commentary is not to condemn well-intentioned authors but to highlight common problems that may not be immediately obvious to a wider readership in the hope of reducing bias, improving patient welfare and influencing the future conduct and reporting of clinical trials on acne. The problems highlighted in this commentary are not restricted to acne trials and we hope that the examples given will help to provide further evidence for the need to improve standards in the reporting of all clinical trials.

## Discussion

From the 25 RCTs found for the 2009 Annual Evidence Update, at least 12 major problems of trial reporting were identified; these are listed in Table 1 in the order in which the trials appeared in the Update [1].

**Table 1:** Common problems in the reporting of acne trials

Problem	Description	References
1. Insufficient power	Underpowered trials can produce false negative results in superiority studies or incorrect claims of equivalence	Pediatric Dermatology [4]; Indian Journal of Dermatology, Venereology and Leprology [5]
2. Duplicate publication	Publication of the same trial more than once can artificially enhance its impact and distort subsequent meta-analyses	American Journal of Clinical Nutrition [8]; Journal of the American Academy of Dermatology [9]; Contraception [11]; Cutis [12]
3. Incorrect statistical comparison	A "within-groups" comparison from baseline may give positive results when the correct "between-groups" comparison is negative	Archives of Dermatology [15]; Saudi Medical Journal [16]
4. "Salami publication"	Splitting the results from a single trial to produce more than one publication can artificially increase its impact	Journal of Drugs in Dermatology [19,20]
5. Inferiority margin not pre-specified	In non-inferiority studies, lack of a pre-specified inferiority margin means that the margin might have been chosen in retrospect to fit the data	Journal of Drugs in Dermatology [19,20]
6. Two independent trials combined and reported as one	Independent trials should be analysed and reported separately before combination in any subsequent meta-analysis	Cutis [22]; Journal of the American Academy of Dermatology [23]
7. Loss of masking due to trial therapies not considered in "double-blind" trials	Comparators with different physical characteristics or adverse effect profiles can cause loss of participant or investigator masking	European Journal of Dermatology[25]; International Journal of Cosmetic Science [26]
8. Stating P values without publishing outcome data	P values can be misleading without confidence intervals and original outcome data	Indian Journal of Dermatology [27]
9. Failure to account for all randomized participants	Absence of an intention-to-treat analysis raises the possibility of attrition bias due to loss of study participants before the primary endpoint	International Journal of Cosmetic Science [26]
10. Selective outcome reporting	Multiple endpoints, rather than a single primary endpoint, allow "data fishing" in which only the positive outcomes are highlighted	Journal of Drugs in Dermatology [28]
11. Treatment effects statistically significant but clinically insignificant	Highly significant P values may mask a small improvement in disease severity that is insufficient to be of clinical benefit to patients	Journal of Drugs in Dermatology [29]
12. Odds ratios used to exaggerate treatment effect	Odds ratios can be misleadingly large when event rates are high - rate ratios give more understandable results	Contraception [11]

### Lack of power

The first problem identified was RCTs being insufficiently powered to provide evidence of no difference between trial interventions. One study [4], designed to assess the effect of exercise on acne, randomized a total of 30 teenage boys to avoid or perform exercise, and the latter group was further divided into those who showered 1 hour or 4 hours later. The small numbers in the three groups produced very wide confidence intervals that illustrated the underpowered nature of the study. It was reported as a pilot study but a power calculation had been performed. A second study [5] which recruited 60 subjects claimed equivalence between an oral acne therapy and the same treatment in combination with topical agents. However, an equivalence margin was not determined in advance and the equivalence claim was made on the basis of non-significant tests for superiority, a problem frequently encountered in clinical trial reporting [6]. In essence, no evidence of an effect had been misinterpreted as evidence of no effect.

### Duplicate publication

There were two sets of duplicate publications, in which the same trial was published more than once, identified in the 2009 Annual Evidence Update. The first [7] was an additional analysis in a subgroup of patients from a trial on low glycaemic load for treating acne that had already been reported twice. The original duplicate publications [8,9] had been picked up by the 2008 Annual Evidence Update [10]; the papers reported the same trial but failed to cross-reference each other and the journal editors had not been informed. In the second set of duplicate publications, primary efficacy outcomes were presented in one paper [11] without indicating the presence of secondary efficacy outcomes, with the latter then being presented in a second paper four months later [12]. The secondary efficacy variables were similar to the primary variables and showed similar results. We believe that all relevant trial results (especially efficacy results) should be presented in one paper. If there are good reasons to split the results, the seminal index paper should make at least some reference to the measurement of other outcomes and whether there is a plan to publish them elsewhere. Several issues arise from duplicate publication. There could be distortion of any subsequent meta-analysis if the study results are counted twice - such a problem has already arisen with the duplicate publication on low glycaemic load [13]. In addition, journal copyright may be infringed, and multiple articles take up additional journal resources. It has also been demonstrated how duplicate publications result in higher citations [14].

### Testing the wrong thing

Another pitfall that we picked up was the issue in a parallel group study of performing a "within-groups" comparison, rather than the correct "between-groups" analysis of change from baseline. In its abstract, a study that compared a computer presentation with a written information handout stated benefit in favour of the computer approach based on a within-groups comparison, despite a non-significant between-groups comparison in the main article text [15]. Another study of two topical treatments for active acne only performed a within-groups comparison [16], so no account was made for the effect of natural disease history, in particular regression to the mean. Whether such erroneous highlighting of results is deliberate or accidental is unclear - we suggest that it can be a ploy used by authors to try and "save face" in the light of an essentially inconclusive study, especially as some journal editors and clinicians will not spot the lack of a correct between-groups statistic.

### "Salami publication" and absent inferiority margins

"Salami publication" of a clinical trial involves splitting the results from a single trial into several packages that are then published separately and may artificially increase the impact of the study [17]. This issue affected a three-armed parallel groups study registered as a single trial on the *ClinicalTrials.gov *database [18]. Two of the treatment arms were separately compared with the third arm and each comparison was published as a stand-alone trial [19,20], albeit in the same journal supplement. It would have been straightforward to report the results of all three arms in a single publication. Neither publication referenced the other. Another problem with the trial is that it was reported as a non-inferiority study but details of the 15% non-inferiority margin were not stated in the ClinicalTrials.gov register entry, so it is uncertain whether this margin was chosen prospectively or retrospectively. We also found an acne study that compared the same antibiotic at a low dose compared with the standard dose for acne which was essentially a non-inferiority trial, but no non-inferiority margin was specified [21].

### Reporting two independent studies as one

Almost the reverse of duplicate publication is pooling the results of more than one previously unpublished, independent clinical trial in a combined analysis, rather than reporting the results separately. Under such circumstances, the larger, combined analysis could produce a significant result when individually the trials fail to reach significance. Two pairs of RCTs combined in single analyses were spotted in the Annual Evidence Update [22,23]. Results of the individual, independent studies were not presented separately. In both cases these were industry-funded studies of novel topical preparations conducted in North America. It is presumed that two identical RCTs were needed for FDA licensing approval. Whilst it is sometimes appropriate to combine similar studies using a formal meta-analytical approach, we suggest that it is inappropriate to only present combined results in the primary publication of two pivotal RCTs [24].

### Were they really "double-blind"?

In RCTs of topical therapy, particular care is needed to ensure that the comparator preparations closely resemble each other, to prevent loss of participant or investigator masking. In placebo-controlled studies the ideal comparator is the vehicle used for the active treatment, but this is not necessarily possible in head-to-head studies of two active treatments. One trial was reported to be "double-blind" but it compared an acne cream with a gel [25], which would differ in appearance and properties on the skin. Another common reason for loss of blinding in RCTs is a frequent adverse effect associated with one of the treatments and not the other. In topical acne therapy, skin irritation often differs between preparations and this probably caused some loss of blinding in a topical retinoid trial reported to be double-blind [26].

### Absent data and missing patients

Good practice in trial reporting is concerned with providing as much original trial data as possible. Confidence intervals are needed as well as just P values. Unfortunately, one efficacy study failed to provide any trial data and relied on stating P values along with a potentially unrepresentative selection of clinical photographs [27]. Another issue of good practice with RCT reporting is to account for all the patients randomized to prevent attrition bias, with an intention-to-treat analysis and a pre-specified method to deal with missing values. One trial randomized 45 participants but included data for only 30 of them at the final 8 week endpoint; no data or explanation were given about those participants who dropped out of the study [26].

### Data fishing, impressive P values, and "plumped up" odds ratios

There are several ways in which a trial report can make the results appear more impressive than they really are. One of these is to "data fish" amongst a large number of outcomes, rather than focus on a single, pre-specified primary outcome. This was probably the case in an acne trial that displayed only its positive outcomes in the abstract [28]. Another issue is reliance on a statistically significant effect that may be insignificant in clinical practice. An impressive P value of 0.001 was used to justify the efficacy of an acne therapy [29], but this equated to only a modest 11% reduction in the acne lesion count, which probably would not be meaningful to a patient. Finally, use of more impressive sounding odds ratios rather than rate ratios was spotted [11] which will give an overestimate when event rates are frequent [30].

## Conclusion

One of the foundations of evidence-based practice is the availability of high quality evidence on which to base clinical decisions. Although some of the trials found in the Annual Evidence Update were reported to a high standard, around a half contained potentially serious reporting problems and framing biases that could mislead the clinical readership.

Many of the problems outlined in this article could have been avoided by adherence to the CONSORT guidelines [31] and prospective trial registration. CONSORT has provided the gold standard for RCT reporting, and adoption of the guidelines by many, but not all, journals has ensured a standardized method of quality control. The CONSORT list can also be used to aid trial design at the planning stage. Prospective trial registration on a public clinical trials database, or publication of the study protocol, is also very helpful for subsequent users of research to ensure that primary endpoints are stated prospectively. In essence, the study designers are asked to "nail their flag to the mast" in advance in terms of their most important endpoint. Again, adoption of this as a requirement for publication by journals has helped to promote its use.

## Competing interests

The authors declare that they have no competing interests.

## Authors' contributions

DJCG carried out the database searches and identified the relevant randomised controlled trials. JRI and HCW carried out the critical appraisal of trial quality and reporting. JRI took the lead in drafting the paper, with contributions and edits by HCW and DJCG. All authors read and approved the final manuscript.
